# IncobotulinumtoxinA Injection for Treating Children with Idiopathic Toe Walking: A Retrospective Efficacy and Safety Study

**DOI:** 10.3390/toxins14110792

**Published:** 2022-11-13

**Authors:** Mirko Filippetti, Alessandro Picelli, Rita Di Censo, Sabrina Vantin, Pietro Nicola Randazzo, Giorgio Sandrini, Cristina Tassorelli, Roberto De Icco, Nicola Smania, Stefano Tamburin

**Affiliations:** 1 Department of Neurosciences, Biomedicine and Movement Sciences, University of Verona, 37100 Verona, Italy; 2 Canadian Advances in Neuro-Orthopedics for Spasticity Congress (CANOSC), Kingston, ON K7K 1Z6, Canada; 3 Department of Brain and Behavioral Sciences, University of Pavia, 27100 Pavia, Italy; 4 Headache Science & Neurorehabilitation Center, IRCCS Mondino Foundation, 27100 Pavia, Italy

**Keywords:** botulinum toxins, pediatrics, rehabilitation, walking

## Abstract

There is no gold-standard treatment for idiopathic toe walking (ITW). Some previous evidence suggested that botulinum neurotoxin-A injection might improve ITW. This is a single-center retrospective study on children with ITW treated with incobotulinumtoxinA injection in the gastrocnemius medialis/lateralis muscles. We screened the charts of 97 ITW children treated with incobotulinumtoxinA (January 2019–December 2021), and the data of 28 of them, who satisfied the inclusion/exclusion criteria, were analyzed. The maximal passive ankle dorsiflexion (knee extended) was assessed at three time points, i.e., immediately before incobotulinumtoxinA injection (T0), after incobotulinumtoxinA injection during the timeframe of its effect (T1), and at follow-up, when the effect was expected to disappear (T2). The maximal passive ankle dorsiflexion was improved by incobotulinumtoxinA injection, and the effect lasted up to 6 months in some children. No adverse effects were reported to incobotulinumtoxinA injections. The treatment with incobotulinumtoxinA might improve the maximal passive ankle dorsiflexion and is safe and well-tolerated in ITW with a longer-than-expected effect in comparison to cerebral palsy. These results may offer ground to future randomized controlled trials and studies assessing the effect of BoNT-A in combination with other non-invasive approaches and exercise programs in children with ITW.

## 1. Introduction

Idiopathic toe walking (ITW) is defined as the absence of heel contact at the initial contact phase of the gait cycle that lasts over the third year of age without known anatomical deformity or neurological deficits and makes the heel strike impossible [1,2,3]. ITW is thus a diagnosis of exclusion of other musculoskeletal or neurological conditions. This pattern of gait was first described in 1967 and termed as “congenital short tendon calcaneus” [2]. Later, the name was changed to “habitual toe walking”, and the term “idiopathic toe walking” was introduced in 1980 [4,5,6]. The prevalence of ITW has been seldom explored, but this walking pattern is estimated to occur in approximately 1 out of 20 children, with a higher prevalence in boys than girls [7,8]. When the children begin to stand and walk, the usual pattern of initial contact is flatfoot walking, and only around the age of 3 do they reach a heel-toe stride [9].

The pathophysiology of ITW is still not known [1]. Increased prevalence of developmental delays and neurodevelopmental diagnoses has been reported in children with ITW [1]. Autism spectrum disorder and language impairment are over-represented in children with ITW [1].

Toe walking can also be secondary to medical conditions such as: (a) neuromuscular changes in the muscle length, innervation, or strength; (b) traumatic or biomechanical changes in the skeletal framework of the pelvis and/or lower limb; (c) neurogenic influences that result in a toe-walking gait [3]. The differential diagnosis between mild cerebral palsy (CP) and ITW is of great importance but can sometimes be difficult due to similarities in walking patterns between the two conditions [10]. There are some pinpoints that may help the clinician to differentiate between CP and ITW. CP is characterized by spasticity, increased hamstring tightness, and coactivation of the gastrocnemius medialis (GM) and gastrocnemius lateralis (GL) muscles during resisted seated knee extension and quadriceps set [10]. CP and ITW are similar in terms of passive ankle dorsiflexion, but hamstring passive range of motion is reduced in CP [10]. Gait analysis may be helpful to differentiate CP and ITW. ITW is characterized by normal hip and knee kinematics during walking, while children with CP show increased hip flexion during all phases of walking and maintained knee flexion during initial contact in stance [10]. Further, ITW typically shows active plantarflexion during swing, striking of the ground in midfoot or forefoot stance during initial contact, and normal knee kinematics, while CP is characterized by active dorsiflexion during the swing phase and striking of the ground in midfoot or forefoot stance during initial contact concurrent with atypical knee kinematics [10]. Moreover, children with ITW show more variability in walking kinematics between trials in comparison to those with mild CP [10].

Botulinum neurotoxin-A (BoNT-A) is widely used to reduce calf muscle spasticity and tightness in children with CP and a toe-walking gait pattern [11,12]. Currently, botulinum neurotoxin has not been approved for children with ITW. To date, the evidence on the effect of BoNT-A injection on ITW is scant and there are no data on incobotulinumtoxinA (Xeomin, Merz, Frankfurt am Main, Germany) for this condition. The aim of this retrospective chart review study is to explore the efficacy and the tolerability of incobotulinumtoxinA for the treatment of children with ITW.

## 2. Results

We screened the charts of 97 children treated with incobotulinumtoxinA from January 2019 to December 2021. Twenty-eight of them satisfied the inclusion and exclusion criteria and were therefore included in the analysis. Baseline demographic and clinical data are reported in Table 1.

Figure S1 shows the study design and the assessment timeline (see Section 5 below for more details).

The maximal passive ankle dorsiflexion at three assessment time points (T0, T1, T2) and the differential maximal passive ankle dorsiflexion between the time points (Δ T1–T0; Δ T2–T0) are reported in Table 2. The maximal passive ankle dorsiflexion was significantly improved at T1 vs. T0 on both sides and persisted as significantly better at T2 vs. T0 on the right side, while it was slightly but not significantly better at T2 vs. T0 on the left side (Table 2).

No adverse effects were reported to any incobotulinumtoxinA injection, except for transient light soreness at the site of injection in 21 patients.

## 3. Discussion

The present data show that (a) the treatment with incobotulinumtoxinA significantly improves maximal passive ankle dorsiflexion in children with ITW [2,13,14], and (b) the effect may last up to six months after incobotulinumtoxinA injection.

Overall, our findings are in keeping with two open studies on children with ITW undergoing onabotulinumtoxinA injection [15,16].

Despite being preliminary and derived from a retrospective analysis, our findings may add to the potential armamentarium for ITW treatment, a field of uncertainty. A recent Cochrane review on this topic yielded inconclusive findings, in that the strength of evidence was too low and the outcome data were too limited when comparing serial casting, BoNT-A, footwear, exercises, and different types of orthoses as interventions [6]. Because of the lack of robust evidence, whether casting, ankle foot orthosis, education towards normal gait, BoNT-A, or a combination of these treatments may affect the natural history of ITW, or merely speed up the improvement of toe-walking gait in some children who would have recovered spontaneously, is still not known [17]. Some authors have proposed surgical approaches to ITW, e.g., percutaneous lengthening of the Achilles tendon [17].

IncobotulinumtoxinA injection into the GM and GL muscles is widely used for the treatment of lower limb spasticity in combination with adjuvant approaches to boost BoNT-A’s effect [18]. These adjuvant modalities have never been tested in ITW [6].

In our cohort, the follow-up assessment, which sometimes coincided with the reinjection visit, was quite delayed (median value more than six months after the first injection). This long distance of follow-up was due to the coexisting COVID-19 pandemic during the study time window that impeded and/or reduced the access of patients to our Neurorehabilitation Unit and delayed the visits in comparison to usual care standards. These figures are in keeping with a study on the impact of the national lockdown due to the COVID-19 pandemic in Italy that reported a delay of BoNT injection by nine months on average in patients with CP [19]. Despite such a long distance from injection, we documented a still-persistent improvement of maximal passive ankle dorsiflexion at T2, which reached statistical significance in comparison to T0 on the right side. This unexpected delay might have uncovered a long-lasting effect of incobotulinumtoxinA injection exceeding the traditional 12-16 weeks. This finding is in keeping with a study on incobotulinumtoxinA in CP, where the most frequently treated muscles were GM and GL, and the reinjection time delay was around six months [20]. Other explanations include the possibility of a partial spontaneous amelioration [17], the paraphysiological nature of ITW [1], and the involvement of neuroplastic changes in the motor system, which have been previously demonstrated after BoNT-A injection and may have exceeded its pharmacokinetics [21].

The importance of the present findings lies in the possibility that the observed improvement induced by incobotulinumtoxinA injection may be associated with multiple positive biomechanical changes, such as the amelioration of the ankle, knee, and hip dynamics, the prevention of abnormal wear and tear on joint articular facets, and the better absorption of the shocks resulting in a more physiological gait attitude [3].

No significant adverse effects were reported, in keeping with the safety profile of incobotulinumtoxinA injection in the calf muscles in patients with CP [12,16,20].

Limitations of this study include the retrospective design, the lack of a control group, the delayed timing of some evaluations, the absence of a time curve of the therapeutic effect, the absence of data on gait analysis, and other outcomes, e.g., patient reported outcomes. Indeed, the maximal passive ankle dorsiflexion suggests a better heel strike but is not direct evidence of it and requires gait analysis confirmation.

## 4. Conclusions

Our data suggest that the treatment with incobotulinumtoxinA improves maximal passive ankle dorsiflexion in ITW and is safe and well-tolerated. These findings also suggest a longer-than-expected effect of incobotulinumtoxinA injection in the GM and GL muscles in children with ITW.

Though preliminary, the present findings offer ground to future randomized controlled trials versus placebo or versus the combination with other non-invasive approaches and exercise programs to improve the walking pattern in children with ITW [15].

## 5. Materials and Methods

This single-center retrospective study analyzed charts of consecutive children with ITW treated with incobotulinumtoxinA at the Neurorehabilitation Unit of the University Hospital of Verona, Italy from January 2019 to December 2021.

Inclusion criteria were: (a) age 3–18 years, (b) diagnosis of ITW, (c) absence of clinical features or spasticity and/or spastic gait pattern, (d) treatment with incobotulinumtoxinA. Exclusion criteria were: (e) diagnosis of CP and/or other disease that could explain the toe-walking gait, (f) previous surgical treatment for ITW, (g) concomitant treatment for ITW (e.g., serial casting), (h) other neurological and/or orthopedic conditions affecting the lower limb and not related with ITW that may have contributed and/or interfered with the ITW gait pattern.

All participants were outpatients. All patients were minors, and, for all of them, parents provided informed consent, which included consent for data extraction from chart reviews, as needed. The study was carried out according to the principles of the Declaration of Helsinki and was approved by the local Review Board (*Comitato Etico per la Sperimentazione Clinica delle Province di Verona e Rovigo*; date of approval: September 7th, 2022; approval number: 3954CESC).

### 5.1. Clinical Evaluation

During the evaluation, patients remained in the supine position with their knees extended. The maximal passive ankle dorsiflexion was measured using a handheld goniometer. The sensitivity of the measurement was set at 5°. The dorsiflexion angle was defined as positive, taking 0° as the neutral position of the joint [22]. The goniometer was positioned with the fulcrum at the ankle and the distal branch was adjusted to the first metatarsal head and the proximal one to the tibial axis. The modified Ashworth scale (MAS) is a 6-point scale that grades the resistance of a relaxed limb to rapid passive stretch (0 = no increase in muscle tone; 1 = slight increase in muscle tone at the end of the range of motion; 1 + = slight increase in muscle tone through less than half of the range of motion; 2 = more marked increase in muscle tone through most of the range of motion; 3 = considerable increase in muscle tone; 4 = joint is rigid) [23]. The MAS was used to exclude patients with spastic calf muscles.

### 5.2. Botulinum Toxin Injection Procedure

Botulinum toxin injection was performed with the patient prone with the foot lying over the edge of the medical examination couch. IncobotulinumtoxinA vials of 100U were reconstituted with 1 ml of physiological solution using 1 mL syringes with a 25 G/16 mm needle or 22 G/32mm needle. The incobotulinumtoxinA total dose and body-weight-corrected dose were below the maximum doses suggested for pediatric patients with spastic CP [24] and were equally divided between the GM and GL muscles of both legs. The GM and GL muscles were injected at the mid-belly in one site under ultrasound (US) guidance after skin disinfection with a 2% electrolytic chlorioxidant solution. The choice of injecting GM and GL muscles was based on kinetic and kinematic differences between physiological and toe-walking strides [25]. The US real-time B-mode ultrasonography MyLab 70 XVision system (Esaote SpA, Genoa, Italy) was used with a linear probe (scanning frequency 13 MHz), which was positioned perpendicular to the affected calf surface. GM and GL muscles were scanned up and down using the “elevator technique”, with the probe placed gently over the skin using a water-soluble transmission gel [22,26,27]. IncobotulinumtoxinA was injected two or more times, but here we report only outcomes after the first injection (Figure S1).

### 5.3. Study Design, Outcome Measures, Adverse Events, and Assessment Times

The maximal passive ankle dorsiflexion was assessed at three time points, i.e., immediately before incobotulinumtoxinA injection (T0), after incobotulinumtoxinA injection during the timeframe of its effect (T1), and at follow-up, when the effect was expected to disappear (T2; Figure S1). Adverse events were recorded by parents in a checklist that listed those most reported (e.g., fever, fatigue, general/local muscle weakness, general/local pain, diarrhea, local soreness, ecchymosis, etc.) and a free space for reporting any additional adverse event [12]. Furthermore, the presence of adverse effects was checked at T1 and T2 visits.

### 5.4. Statistical Analysis

Statistical analysis was carried with the IBM SPSS version 20.0 package. The normality of variables distribution was analyzed with the Skewness-Kurtosis test. The Wilcoxon signed rank test was used for the comparison of maximal passive ankle dorsiflexion at different time points. *p* < 0.05 (two-tailed) with Bonferroni’s correction for multiple comparisons was taken as the significance threshold.

## Figures and Tables

**Table 1:** Baseline demographic and clinical data.

Variables	Values ^†^
Age (years; mean ± SD)	8.3 ± 3.1
Weight (Kg; mean ± SD)	33.5 ± 13.9 kg
Sex (male/female)	22/6
Dominant side (right/left)	18/4
Number of injections (median, IQR)	2, 2–3
Distance between T1 and T0 assessment (days; median, IQR)	45, 33–54
Distance between T2 and T0 assessment (days; median, IQR)	198, 133–287
IncobotulinumtoxinA dose (units; mean ± SD)	171.4 ± 66.1
IncobotulinumtoxinA dose/Kg (units/Kg; mean ± SD)	6.6 ± 2.2

^†^ Data are reported as mean ± SD when the distribution is normal, otherwise as mean, interquartile range (IQR).

**Table 2:** Outcome measures.

Maximal Passive Ankle Dorsiflexion (°)	Values ^†^	*p* Value
*Right side*		
T0 (immediately before injection)	−3.2 ± 6.4	
T1 (after injection)	3.0 ± 5.6	<0.001 ^‡,^*
T2 (follow-up)	1.2 ± 7.4	0.023 ^¶,^*
Δ maximal passive ankle dorsiflexion (T1–T0)	6.7 ± 6.3	
Δ maximal passive ankle dorsiflexion (T2–T0)	3.6 ± 6.7	
*Left side*		
T0 (immediately before injection)	−2.9 ± 6.5	
T1 (after injection)	2.4 ± 7.1	0.001 ^‡,^*
T2 (follow-up)	−1.0 ± 8.2	0.037 ^¶^
Δ maximal passive ankle dorsiflexion (T1–T0)	6.1 ± 5.8	
Δ maximal passive ankle dorsiflexion (T2–T0)	1.2 ± 7.1	

^†^ Data are reported as mean ± SD. * Significant comparison (*p* < 0.05, two-tailed, with Bonferroni’s correction). ^‡^ T1 vs. T0 comparison (Wilcoxon signed rank test). ^¶^ T2 vs. T0 comparison (Wilcoxon signed rank test).

## Data Availability

Original data are available by contacting the corresponding authors upon reasonable request.

## Supplementary Materials

The following supporting information can be downloaded at: https://www.mdpi.com/article/10.3390/toxins14110792/s1, Figure S1. Timeline of the outcome measures assessment and the incobotulinumtoxin injection. Δ maximal passive ankle dorsiflexion (T1–T0) = maximal passive ankle dorsiflexion at T1—maximal passive ankle dorsiflexion at T0; Δ maximal passive ankle dorsiflexion (T2–T0) = maximal passive ankle dorsiflexion at T2—maximal passive ankle dorsiflexion at T0.

## References

[1] Engström P., Tedroff K. (2018). Idiopathic Toe-Walking: Prevalence and Natural History from Birth to Ten Years of Age. J. Bone Jt. Surg. Am..

[2] Hall J.E., Salter R.B., Bhalla S.K. (1967). Congenital short tendo calcaneus. J. Bone Jt. Surg. Br..

[3] Williams C.M., Tinley P., Rawicki B. (2014). Idiopathic toe-walking: Have we progressed in our knowledge of the causality and treatment of this gait type?. J. Am. Podiatr. Med. Assoc..

[4] Griffin P.P., Wheelhouse W.W., Shiavi R., Bass W. (1977). Habitual toe-walkers. A clinical and electromyographic gait analysis. J. Bone Jt. Surg. Am..

[5] Conrad L., Bleck E.E. (1980). Augmented auditory feedback in the treatment of equinus gait in children. Dev. Med. Child. Neurol..

[6] Caserta A.J., Pacey V., Fahey M., Gray K., Engelbert R.H., Williams C.M. (2019). Interventions for idiopathic toe walking. Cochrane Database Syst. Rev..

[7] Engelbert R., Gorter J.W., Uiterwaal C., van de Putte E., Helders P. (2011). Idiopathic toe-walking in children, adolescents and young adults: A matter of local or generalised stiffness?. BMC Musculoskelet. Disord..

[8] Engström P., Tedroff K. (2012). The prevalence and course of idiopathic toe-walking in 5-year-old children. Pediatrics.

[9] Ivanenko Y.P., Dominici N., Lacquaniti F. (2007). Development of independent walking in toddlers. Exerc. Sport Sci. Rev..

[10] Schlough K., Andre K., Owen M., Adelstein L., Hartford M.C., Javier B., Kern R. (2020). Differentiating Between Idiopathic Toe Walking and Cerebral Palsy: A Systematic Review. Pediatr. Phys. Ther..

[11] Ade-Hall R.A., Moore A.P. (2000). Botulinum toxin type A in the treatment of lower limb spasticity in cerebral palsy. Cochrane Database Syst. Rev..

[12] Carraro E., Trevisi E., Martinuzzi A. (2016). Safety profile of incobotulinum toxin A (Xeomin^®^) in gastrocnemious muscles injections in children with cerebral palsy: Randomized double-blind clinical trial. Eur. J. Paediatr. Neurol..

[13] Brouwer B., Davidson L.K., Olney S.J. (2000). Serial casting in idiopathic toe-walkers and children with spastic cerebral palsy. J. Pediatr. Orthop..

[14] Williams C.M., Tinley P., Curtin M. (2011). The Toe Walking Tool: A novel method for assessing idiopathic toe walking children. Gait. Posture.

[15] Engström P., Gutierrez-Farewik E.M., Bartonek A., Tedroff K., Orefelt C., Haglund-Åkerlind Y. (2010). Does botulinum toxin A improve the walking pattern in children with idiopathic toe-walking?. J. Child. Orthop..

[16] Brunt D., Woo R., Kim H.D., Ko M.S., Senesac C., Li S. (2004). Effect of botulinum toxin type A on gait of children who are idiopathic toe-walkers. J. Surg. Orthop. Adv..

[17] Dietz F., Khunsree S. (2012). Idiopathic toe walking: To treat or not to treat, that is the question. Iowa Orthop. J..

[18] Picelli A., Filippetti M., Sandrini G., Tassorelli C., De Icco R., Smania N., Tamburin S. (2021). Electrical Stimulation of Injected Muscles to Boost Botulinum Toxin Effect on Spasticity: Rationale, Systematic Review and State of the Art. Toxins.

[19] Tarantino D., Gnasso R., Migliore F., Iommazzo I., Sirico F., Corrado B. (2021). The effects of COVID-19 pandemic countermeasures on patients receiving botulinum toxin therapy and on their caregivers: A study from an Italian cohort. Neurol. Sci..

[20] León-Valenzuela A., Palacios J.S., Del Pino Algarrada R. (2020). IncobotulinumtoxinA for the treatment of spasticity in children with cerebral palsy—A retrospective case series focusing on dosing and tolerability. BMC Neurol..

[21] Hok P., Veverka T., Hluštík P., Nevrlý M., Kaňovský P. (2021). The Central Effects of Botulinum Toxin in Dystonia and Spasticity. Toxins.

[22] Picelli A., Bonetti P., Fontana C., Barausse M., Dambruoso F., Gajofatto F., Girardi P., Manca M., Gimigliano R., Smania N. (2012). Is spastic muscle echo intensity related to the response to botulinum toxin type A in patients with stroke? A cohort study. Arch. Phys. Med. Rehabil..

[23] Bohannon R.W., Smith M.B. (1987). Interrater reliability of a modified Ashworth scale of muscle spasticity. Phys. Ther..

[24] Heinen F., Kanovský P., Schroeder A.S., Chambers H.G., Dabrowski E., Geister T.L., Hanschmann A., Martinez-Torres F.J., Pulte I., Banach M. (2021). IncobotulinumtoxinA for the treatment of lower-limb spasticity in children and adolescents with cerebral palsy: A phase 3 study. J. Pediatr. Rehabil. Med..

[25] De Pieri E., Romkes J., Wyss C., Brunner R., Viehweger E. (2022). Altered Muscle Contributions are Required to Support the Stance Limb During Voluntary Toe-Walking. Front. Bioeng. Biotechnol..

[26] Picelli A., Baricich A., Chemello E., Smania N., Cisari C., Gandolfi M., Cinone N., Ranieri M., Santamato A. (2017). Ultrasonographic Evaluation of Botulinum Toxin Injection Site for the Medial Approach to Tibialis Posterior Muscle in Chronic Stroke Patients with Spastic Equinovarus Foot: An Observational Study. Toxins.

[27] Filippetti M., Di Censo R., Varalta V., Baricich A., Santamato A., Smania N., Picelli A. (2022). Is the Outcome of Diagnostic Nerve Block Related to Spastic Muscle Echo Intensity? A Retrospective Observational Study on Patients with Spastic Equinovarus Foot. J. Rehabil. Med..

