# Supplementary material for: IncobotulinumtoxinA Injection for Treating Children with Idiopathic Toe Walking: A Retrospective Efficacy and Safety Study
Source: Toxins (Basel). 2022 Nov 13;14(11):792. doi: 10.3390/toxins14110792 (PMC9694855; doi:10.3390/toxins14110792)
Supplement: Supplementary file 1 [file toxins-14-00792-s001.zip › toxins-1953855-supplementary.pdf]

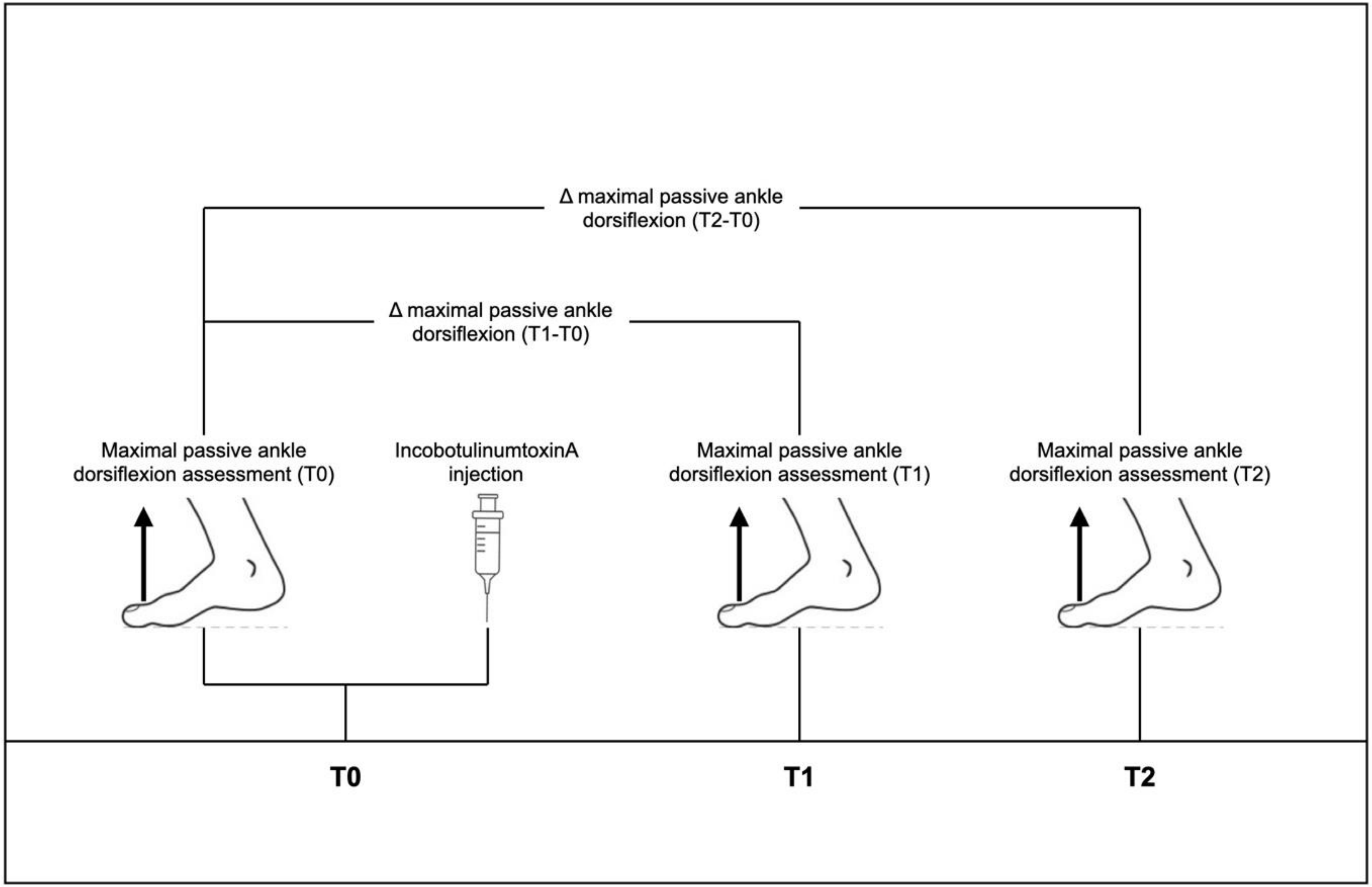

Supplementary Figure S1. Timeline of the outcome measures assessment and the incobotulinumtoxin injection. Δ maximal passive ankle dorsiflexion (T1-T0) = maximal passive ankle dorsiflexion at T1 – maximal passive ankle dorsiflexion at T0; Δ maximal passive ankle dorsiflexion (T2-T0) = maximal passive ankle dorsiflexion at T2 – maximal passive ankle dorsiflexion at T0.
